# The Hunt for Proteins in Dinosaur Fossils

**DOI:** 10.1021/acscentsci.5c01027

**Published:** 2025-06-17

**Authors:** Carolyn Wilke

## Abstract

Reports of peptide sequences in 2005 have ignited controversy and interest in paleoproteomics.

## Graphical abstract

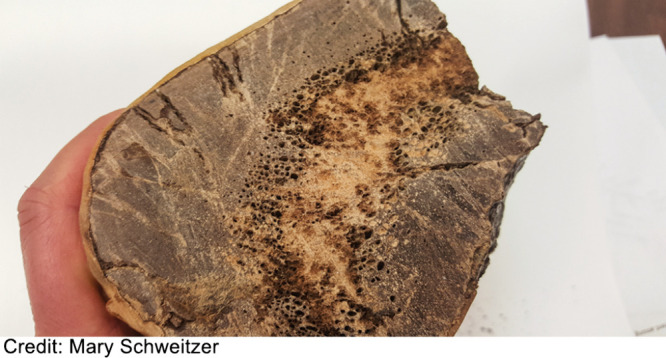

In 2003, molecular paleontologist Mary Schweitzer received a palm-sized chunk of a femur from a *Tyrannosaurus rex* fossil. She looked at the sample, unearthed from the Hell Creek formation in Montana, and remarked to her lab technician, “Oh my gosh—we have a girl, and it’s pregnant,” Schweitzer recalls. “She looked at me like I had lost my mind, which most people do.”

Over the following years, Schweitzer’s work on that specimen produced extraordinary reports. The cause of her proclamation was what appeared to be medullary bone in the fossil. In female birds, this layer forms during the egg-laying cycle to provide a repository of calcium for eggshells.

But there were more surprises from the *T. rex* sample. When the scientists removed the hard mineralized bone, they were left with a soft, stretchy organic material. “Nobody ever thought the organics in bone would last 65 million years,” says Schweitzer, now a professor emeritus at North Carolina State University.

In a 2005 *Science* paper, Schweitzer’s team reported transparent, flexible structures in the fossil that appeared to be blood vessels, some containing dark red spheres that looked like blood cells. Those structures had to be made of something, possibly proteins, Schweitzer reasoned, launching her quest to recover remnants of original proteins from the *T. rex*. In 2007 in *Science*, her team published peptide sequences, including seven from collagen, from the *T. rex* bone—the first-ever proteomics for an ancient sample.

The report sparked a debate about the find’s authenticity. “It’s become so iconic,” says Matthew Collins, an archeological scientist at the University of Copenhagen and the University of Cambridge. “It’s never been proven. It’s never been stamped down.” Collins is not involved with Schweitzer’s work and has studied ancient proteins for decades.

Earlier this year, another group published collagen sequences from a different Hell Creek dinosaur fossil. Their proteomic analysis of bone from a duck-billed dinosaur called *Edmontosaurus* found sequences that overlap with those reported by Schweitzer for the *T. rex* and with sequences reported in 2009 for a relative of *Edmontosaurus* called *Brachylophosaurus canadensis* .

**Figure:**
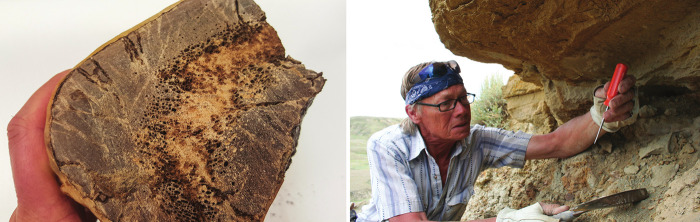
Molecular paleontologist Mary Schweitzer received a chunk of a *Tyrannosaurus rex* femur from late paleontologist Bob Harmon (pictured, right image), who found the fossil in the side of a cliff in Montana. The sample (left image)—called *B. rex* in honor of Harmon—led to a number of *Science* papers, including one reporting peptide sequences for the ancient beast. Credit: Mary Schweitzer.

“This is potentially a really significant study because it’s the first independent replication of the sequence,” Collins says. Still, some scientists—Collins included—are cautious.

The early reports of dinosaur collagen ignited greater interest in proteomes of the past. Ancient proteins, or what’s left of them, can reveal clues about long-gone life-forms, from specific adaptations to evolutionary relationships. Researchers have uncovered processes that act on fossils in the earth to transform biomolecules and potentially preserve them. As the field advances, scientists continue to explore the chemistry—that in the laboratory and that which has occurred across the ages—that may unearth insights from proteomes of the distant past.

## How long can collagen last?

In their search for leftover proteins, many researchers homed in on collagen because of its abundance and relative stability. Collagen occurs in all multicellular animals and is highly conserved across evolution. In bone, collagen makes up 90% of organic material. There, tiny plates of the bone mineral bioapatite constrain collagen fibers to keep them from spreading apart, which would quicken their degradation.

**Figure:**
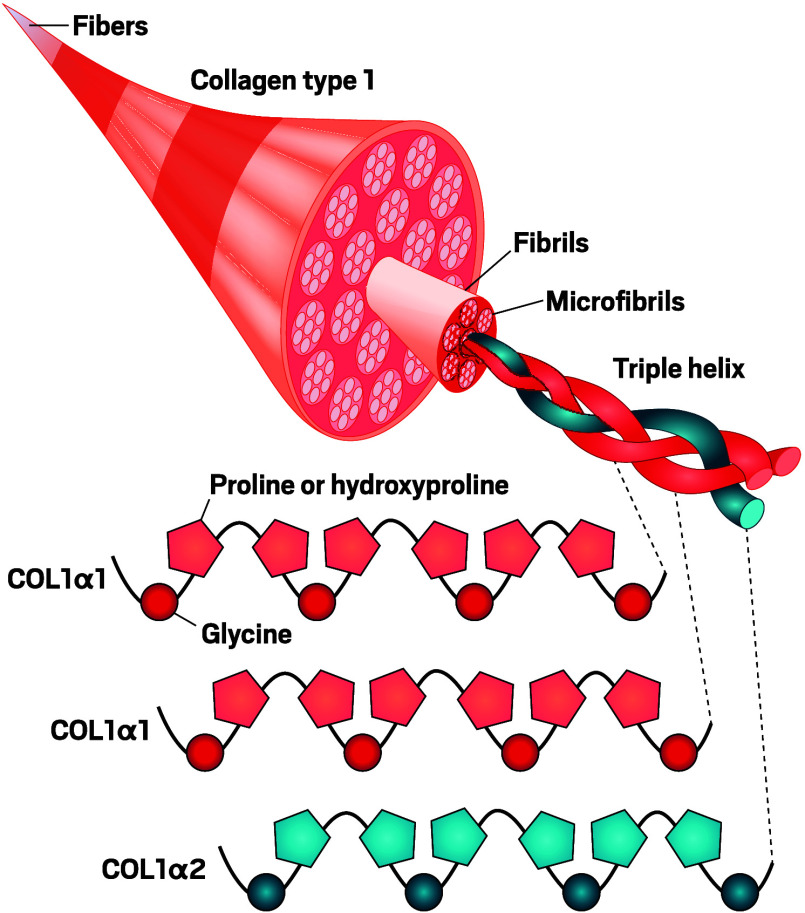
Collagen’s long amino acid strands form triple helices, which band together into larger fibers that give animal tissues their structure. A key characteristic of collagen sequences is that every third amino acid is a relatively compact glycine, which allows tight turns perfect for forming helices. In bones, tiny crystals of bioapatite keep collagen microfibrils from spreading apart. In ancient samples, potential damage to the structure at levels from the fibers to the sequences may pose challenges to collagen preservation. Credit: Adapted from *Proc. Natl. Acad. Sci. U.S.A.* (used under CC BY 4.0).

But it’s not clear how long collagen can stick around. Some of the earliest doubts about the dinosaur collagen sequences hinged around models of collagen decay.

Around 2 decades ago, Collins’s then student Colin Smith spent years degrading bone collagen at low temperatures—“the world’s most boring PhD,” as Collins refers to it. Smith and Collins worked out how temperature affected collagen degradation in water. Smith also plotted the ages of bones in which collagen had been found against the temperatures they had endured. With few exceptions, the ages of the collagen-containing bones fit Smith’s temperature-degradation model.

All together, the work suggested that at temperatures above 20 °C—as occurred in the past in the Hell Creek formation where the *T. rex* bones were found—fossils wouldn’t yield detectable collagen after 15,000 years, let alone 68 million years.

On the other hand, there have been reports of collagen beating the heat. Carli Peters, an archeological scientist at the University of Algarve, and her colleagues surveyed protein preservation in fossils from a range of Australian environments. Their study sites included a limestone cave in northeastern Australia—a subtropical environment where the average temperature is a warm 30 °C.

“We really didn’t expect anything to be preserved,” Peters says. But several fossils from the cave showed collagen preservation in bones dating to some 70,000 years ago. The team looked for signs of contamination. But all the collagens that the researchers turned up were from ancient Australian fauna, some that went extinct around 50,000 years ago.

It is not clear how those molecules persisted when bone collagens at cooler sites did not. Many factors—soil chemistry, microbial activity, burial conditions, the mineral environment—influence whether biomolecules in a fossil can stick around, Peters says.

One idea is that over time, environmental minerals lock the bioapatite into place, protecting the collagen from degrading. Perhaps such a mechanism could help preserve collagen across an even longer time scale.

## Covered by cross-linking

In roughly the past decade, researchers have surfaced a suite of reactions that might protect fossil proteins from the ravages of time, including from the thermal degradation reported by Collins and Smith. These new studies report the same type of chemical transformation: cross-linking, says Jasmina Wiemann, a molecular paleobiologist at Johns Hopkins University.

In this process, biomolecules form links between each other to increase their stability. Although researchers have observed cross-linked fossil proteins, they are still working out what conditions trigger these reactions.

In 2014, Schweitzer’s team proposed that cross-linking chemistry may have preserved ancient tissue in dinosaur fossil samples, including in the pregnant *T. rex* they reported collagen from. The team suggested that iron particles, formed after the breakdown of iron-containing biomolecules, might spur Fenton chemistry that creates free radicals that bash into proteins and haphazardly connect them together.

Similarly, Wiemann and colleagues have found that oxygen-containing radicals can turn protein strands into a 3D mesh. Its components are somewhat resistant to being chewed up by microbes because cross-linking tends to knock out sites that protein-cutting enzymes target to snip.

Wiemann notes that cross-linking is useful for more than just preservation; it can help identify promising fossils to analyze. Cross-linking forms chromophore groups and makes fossils appear darker to the naked eye, so Wiemann’s team uses what she calls “the chocolate color gradient” when looking for fossils: samples with hues ranging from those of 45% cocoa milk chocolate to 99% cocoa dark chocolate in a light-colored sediment. “Most of the time, fossil bones in these colors have outstanding molecular preservation and high abundances of organics,” she says.

## An antibody bind

With ancient samples, the specter of contamination always looms. Critics of the Schweitzer’s 2007 *Science* paper have invoked contamination from modern materials or even microbes that have infiltrated Cretaceous fossils.

Some have found fault with the studies’ analyses, while others found no flaws. The discussion has left the archeological protein community with reams of papers and plenty of questions.

One such question is whether some methods for detecting the collagen were appropriate. Several of Schweitzer’s studies use a technique called immunohistochemistry staining, which relies on antibodies to reveal the presence of a particular protein. “The choice of method was what got her a lot of very skeptical feedback,” Wiemann says.

When this method works, antibodies chosen by the researcher selectively latch on to a molecule of interest—and only that molecule—and confirm its presence. But sometimes antibodies glom on to nontarget molecules, giving a false positive result. Schweitzer’s team has controlled for this possibility in a variety of ways over the years. “I don’t know how you can still say that this is a spurious finding,” she says.

Fossil samples provide extra challenges: the proteins they contain may not be well documented, so the perfect antibody for an assay might not be known. Schweitzer’s immunohistochemistry studies used collagen antibodies from modern birds, but dinosaur collagen may differ too much from bird collagen to reliably interact with bird antibodies. Plus, genetics aside, the probability of these large biomolecules staying intact over millions of years without chemical alteration “is pretty much zero,” Wiemann says.

Researchers have studied whether collagen’s propensity to bind certain antibodies can persist after millennia. There is “a good amount of data” supporting the idea that these ancient collagen remnants may maintain a morphology that could allow for specific antibody binding, Wiemann says. But whether the antibodies used are 100% specific for these collagens, “it is still impossible to say.”

## ‘Case not proven’

Now there’s a new analysis of a fossil from the same formation as Schweitzer’s *T. rex* and *B. canadensis.* Recently, mass spectrometry experts got their hands on an *Edmontosaurus* fossil excavated from a part of Hell’s Creek that extends into South Dakota. In January, researchers reported peptide sequences from the hip bone of the large herbivore.

At around 20 kg, “it’s a huge fossil,” says mass spectrometrist Steve Taylor of the University of Liverpool. Taylor’s team scooped out a bit of the bone for a suite of tests.

Researchers often use Fourier-transformed infrared spectroscopy, or FTIR, to look for organics in fossil material. Taylor’s team saw a tiny hump in an interesting part of the fossil’s FTIR spectrum. It is the same region where collagen pops up in a modern-day turkey bone’s FTIR spectrum. Using cross-polarized light microscopy, the researchers saw colorful domains suggesting traces of collagen throughout the bone.

**Figure:**
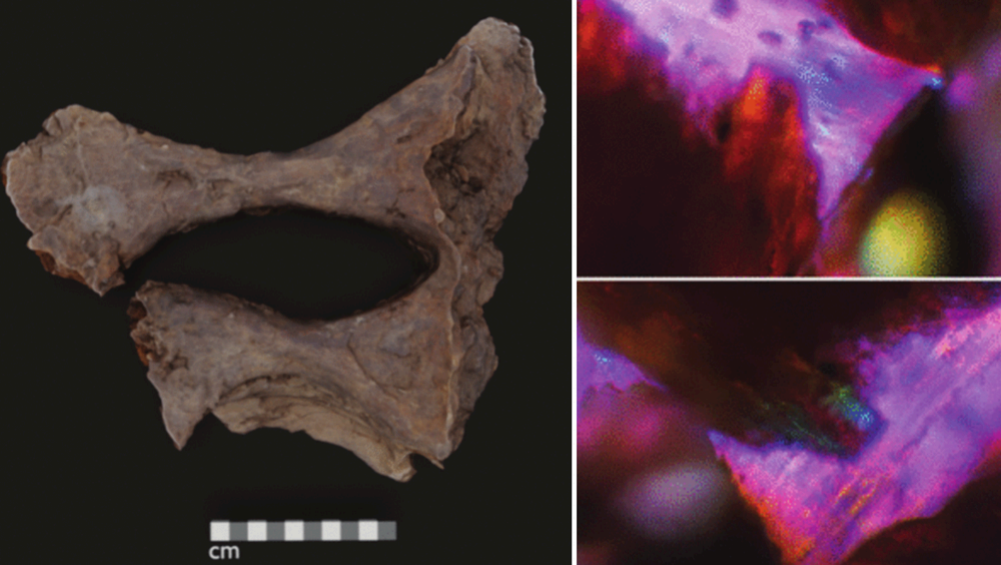
Steve Taylor and his colleagues analyzed an *Edmontosaurus* sacrum (left). Cross-polarized light micrographs show a region that, when rotated, changes colors from orange (top right image, center) to blue-green (bottom right image, center), suggesting the presence of collagen. Credit: Adapted from *Anal. Chem* .

The team reported six collagen peptides, which were all partial overlaps with sequences from other ancient species reported by Schweitzer and colleagues. All six corresponded to sequences for the *B. canadensis*, which belongs to the same family as the *Edmontosaurus*, and three also corresponded to sequences reported for *T. rex*.

This should settle the controversy, Taylor says. “When you’ve got multiple lines of evidence all pointed in the same way,” he says, “you have to go where the science leads.”

Some staunch skeptics are unlikely to change their minds, but others point to lingering questions about the data.

Researchers can look to the sequences themselves for hints about authenticity. For instance, the spread of peptide lengths found by mass spectrometry typically differs in ancient and modern samples. Prior to taking a bottom-up proteomic measurement, researchers typically chop up samples with the enzyme trypsin, which breaks proteins in specific spots. Sequences from fresh samples will primarily show these clean breaks. But old proteins typically have more random breakages, which Collins calls “ragged ends.”

In Schweitzer’s 2007 paper, peptides from a mastodon sample that is hundreds of thousands of years old show these ragged ends. Those of the *T. rex*, however, do not, Collins says. Likewise, Taylor’s *Edmontosaurus* sequences do not have ragged ends. “It’s clean cut like it’s a fresh protein,” Collins says.

“If you’ve only got clean cuts, that means that that sequence is still essentially intact,” Collins says. “And that’s kind of remarkable.”

It is possible that there’s carryover from previous samples run on the analytical equipment, Collins says. Though researchers are careful to run blanks between samples, if they analyzed modern samples first, it may be that bits of fresh samples stuck to the instrument only to be released with ancient samples that might contain harsh reagents used to extract proteins.

But it is also possible that there’s some sort of exceptional preservation that maintained these samples—and that is not an absurd idea, Collins notes. Still though, “at the moment, my view from all the data on Hell Creek is case not proven.”

## Expanding the search

Across the years, Schweitzer has hewed to the approach of disproving—rather than proving—her hypotheses.

This has meant using dozens of techniques to examine different lines of evidence. And it has meant doing analyses even when the results would seem obvious—for instance, looking for biomolecules from the Cretaceous. But it is better to set aside assumptions, she says. “One of the things that kills scientific investigation is conventional wisdom.”

The results Schweitzer published sparked a systematic search for biomolecules in fossils, Wiemann wrote recently in *Nature*. In 2005, collagen was the frontier, Wiemann says. Since then, scientists have branched out to explore other molecules—from keratin to pigments such as melanin.

Peptide sequences might not always be the best bet for gleaning information from the relics of extinct life. Mass spectrometry assesses a molecule’s presence or absence. But it is not ideal for surveying what’s in a complex, altered sample, Wiemann says. Other techniques, such as those based on light spectroscopy, are better at taking a census of the types of organics that have stuck around, Wiemann says, and they provide hints about lifestyle such as metabolism.

And the work on collagen likely is not done yet. Methodological advances could someday bring greater certainty of the authenticity of ancient proteins. Isotope data can tell the age of a sample, but Collins dreams of being able to get this information for individual peptides along with their sequences.

More researchers than ever are taking up the challenges offered by these ancient molecules. “The cool thing for me at the end of my career is how many more people are looking at and recovering proteins from dinosaurs,” Schweitzer says.

*Carolyn Wilke is a freelance contributor to* Chemical & Engineering News *, the independent news outlet of the American Chemical Society.*

